# Large copy number variants are an important cause of congenital hyperinsulinism that should be screened for during routine testing

**DOI:** 10.3389/fendo.2025.1514916

**Published:** 2025-02-18

**Authors:** Sarah E. Flanagan, Isabella-Anna Lazaridi, Jonna M. E. Männistö, Jasmin J. Bennett, Oguzhan Kalyon, Matthew B. Johnson, Matthew N. Wakeling, Jayne A. L. Houghton, Thomas W. Laver

**Affiliations:** ^1^ Department of Clinical and Biomedical Science, University of Exeter, Exeter, United Kingdom; ^2^ Kuopio Pediatric Research Unit (KuPRu), University of Eastern Finland, Kuopio, Finland; ^3^ Exeter Genomics Laboratory, Royal Devon University Healthcare NHS Foundation Trust, Exeter, United Kingdom

**Keywords:** hyperinsulinism, hypoglycemia, copy number variants, deletion, duplication, genetics

## Abstract

**Introduction:**

Congenital hyperinsulinism (HI) is characterized by inappropriate insulin secretion from the pancreatic beta-cells which causes severe hypoglycemia. Copy number variants (CNVs) encompassing multiple genes (contiguous gene CNVs) can cause syndromic forms of HI although they are not typically screened for during routine genetic testing for this condition. We aimed to assess the prevalence of disease-causing contiguous gene CNVs in a cohort of individuals referred for HI genetic testing.

**Methods:**

Our cohort consisted of 3,763 individuals, of which 1,916 had received a genetic diagnosis for their HI and 1,847 were genetically unsolved following routine testing. We screened for 6 different contiguous gene CNVs using next-generation sequencing data from all individuals in the genetically unsolved cohort and searched for patients in our solved cohort who had already been found to have one of these CNVs.

**Results:**

We identified a contiguous gene CNV affecting 5 of the 6 genomic loci in 53 probands; 28 from the solved cohort and 25 from the genetically unsolved cohort. Variants on the X chromosome were most common, being detected in 24/53 children. Overall, these variants represented 2.7% (53/1,941) of genetic diagnoses, which is similar to the prevalence of variants in other commonly screened HI genes.

**Discussion:**

These results confirm that contiguous gene CNVs are an important cause of HI which should be included in standard gene panel testing processes as this will improve pick-up rates for genetic diagnoses in HI.

## Introduction

Congenital Hyperinsulinism (HI) is the most common cause of persistent hypoglycemia in infants and children. In most cases, it results from the disruption of genes that control insulin secretion from the pancreatic beta-cell (1, 2).

Over 30 genetic forms of HI have been described which cause an isolated or syndromic form of the condition (3, 4). Many different types of genetic variation which disrupt the known HI genes/genetic loci are reported. These include single nucleotide variants (SNVs), indels, methylation defects, aneuploidies and large deletions or duplications (referred to as copy number variants; CNVs) (5).

Partial or whole gene deletions and/or duplications have been reported in at least 5 HI disease genes (6–10). Deletions and duplications which encompass multiple genes have also been described in individuals with HI, who typically have a syndromic condition (11–18). These variants are referred to as contiguous gene CNVs.

The molecular mechanism of HI is apparent when the contiguous gene CNV disrupts a known HI gene. For example, in Usher syndrome type 1C, the 122-kb homozygous deletion on chromosome 11p15.1 encompasses *ABCC8* (13, 19), and in the chr20p deletion syndrome, HI results from heterozygous loss of the *FOXA2* gene or its regulatory elements (14). For other contiguous gene CNVs, the critical region for the HI may not have been defined (12, 20) or the deletions may be coincidental or linked to phenotypic risk factors (21).

The size of the disease-causing contiguous gene CNV will vary between individuals, with their phenotype determined by the combination of genes disrupted by the copy number change. Variable penetrance and variable expressivity have also been reported (14, 22, 23). In some children, the presence of syndromic features may raise clinical suspicion of a contiguous gene CNV, prompting genome-wide copy number analyses by karyotyping, microarray or whole genome sequencing (WGS). In contrast, children who present with HI may undergo HI genetic testing as a first-line investigation.

Many genomic laboratories perform targeted analysis of the coding regions of the known HI disease genes during routine screening. While most will employ the necessary bioinformatic pipelines to detect large duplications or deletions affecting a gene on the HI panel, they often do not screen for contiguous gene CNVs where the critical region has not been determined, as these are harder to target and analyze using conventional methods. Consequently, the overall contribution of these variants to the etiology of HI has not been established although some studies have reported the prevalence of individual contiguous gene CNVs in HI cohorts (12, 14, 20).

In this study, we assessed the overall contribution of disease-causing contiguous gene CNVs to the etiology of HI. To do this we performed copy number analysis in a large cohort of patients referred to our laboratory for routine genetic testing for HI.

## Methods

### Participants

We studied an international cohort of 3,763 probands referred to the Exeter Genomics Laboratory for HI genetic testing over a 20-year period (2003-2023). Clinical information was provided at referral using standardized request forms. This included extra-pancreatic features that manifest as part of a syndrome, neurodevelopmental abnormalities, structural or functional organ abnormalities, growth delay, and facial dysmorphism. Seizures were not included as an additional feature in this study as they could be due to hypoglycemia. The study complied with the Declaration of Helsinki with informed consent obtained from all patients or their parents/guardians. Ethical approval for genetic testing was received from the Genetic Beta-Cell Research Bank (517/WA/0327).

The 3,763 patients were grouped into the solved cohort and unsolved cohort. The solved cohort consisted of 1,916 individuals where previous genetic testing by Sanger sequencing, targeted next generation sequencing (tNGS) and/or karyotype/microarray analysis had identified the cause of their HI. The unsolved cohort included 1,847 individuals where disease-causing SNVs and partial/whole gene deletions of a minimum of 11 HI genes (*ABCC8*, *CACNA1D*, *GCK*, *GLUD1*, *HADH*, *HNF1A*, *HNF4A*, *INSR*, *KCNJ11*, *SLC16A1*, and *TRMT10A*) had been excluded by tNGS (24).

### Copy number variant analysis

We identified 6 different contiguous gene CNVs reported to be causative of HI within the literature which had sufficient evidence to support pathogenicity according to the ACMG/ClinGen CNV scoring matrix (25) (**Table 1**). These included deletions at chr5q35.1-q53.3 causing Sotos syndrome, deletions/duplications at chr11p15 causing Beckwith Wiedemann syndrome, chr11p14-p15 deletions causing Usher syndrome type 1c, anomalies on the X chromosome causing Turner syndrome and chr20p11.2 and chr9p24.3-p24.1 deletions. To establish the region of interest for each contiguous gene CNV we took the widest reported boundaries or the minimal critical region when defined (**Table 1**).

**Table 1:** Table detailing the six syndromes screened and their associated genomic regions.

Associated syndrome (OMIM ID)	Chromosome band	Start (GRCh37/hg19)	End (GRCh37/hg19)	Deletion/Duplication	Known HI gene in region	References
Sotos syndrome (#117550)	chr5q35.1-q35.3	168500001	180915260	Deletion	*NSD1*	(35, 36)
Chromosome 9p deletion syndrome (#158170)	chr9p24.3-p24.1	0	7200000	Deletion	*-*	(12)
Beckwith-Wiedemann syndrome (#130650)	chr11p15	2019432	2170796	Deletion/ Duplication	*-*	(37)
Usher syndrome type 1c (#276904)	chr11p14-p15	17432062*	17553089*	Deletion	*ABCC8*	(13)
Chromosome 20p11.2 deletion syndrome	chr20p11.2	20158646	22525896	Deletion	*FOXA2*	(14)
Turner syndrome	chrX	0	155270560	Deletion	*KDM6A*	(11, 20)

Individuals with congenital hyperinsulinism were screened for contiguous gene copy number variants (CNVs) overlapping these genomic coordinates. *As exact coordinates for the Usher syndrome deletion are not provided in the literature we used the genomic coordinates for *ABCC8* exon 22 and *USH1C* exon 19 which are deleted in this condition to define this region.

We screened all 6 contiguous gene CNVs in the 1,847 individuals from the unsolved cohort using read-depth analysis of next-generation sequencing data. When available, WGS data was used in preference to tNGS which relied on the analysis of low-level off-target reads using our in-house software SavvyCNV (methods previously described (26)) (WGS n=178, tNGS n=1,669). To improve specificity, we ran the pipeline in non-mosaic mode. We called variants that extended across the region of interest of the reported variant, except for those on the X chromosome. For this analysis we screened the X chromosomes in all females for deletions >1Mb which encompassed the *KDM6A* gene or where a ring chromosome was predicted.

### SNP array confirmation of suspected deletions of the Usher syndrome type 1c locus

We undertook SNP array genotyping on samples from 8 individuals in the solved cohort with a homozygous deletion of *ABCC8* exons 1-22, which had raised the possibility of an extended deletion and a diagnosis of Usher syndrome type 1C (13, 27, 28). The analysis was performed on an Illumina GSAMD-v3 array by Erasmus MC (Rotterdam, Netherlands). We used PennCNV to call deletions (29). Deletions were excluded if they had a confidence score <20 or were in a low coverage region (<10 probes covering the CNV).

### Methylation analysis of the 11p.15.5 differentially methylated region

When a contiguous gene CNV was identified which extended across the differentially methylated locus at chr11p15.5, we performed methylation-specific multiplex ligation-dependent probe amplification (MS-MLPA) to determine the parental origin of the affected chromosome (MRC-Holland kit ME030-C3).

## Results

**Figure 1:**
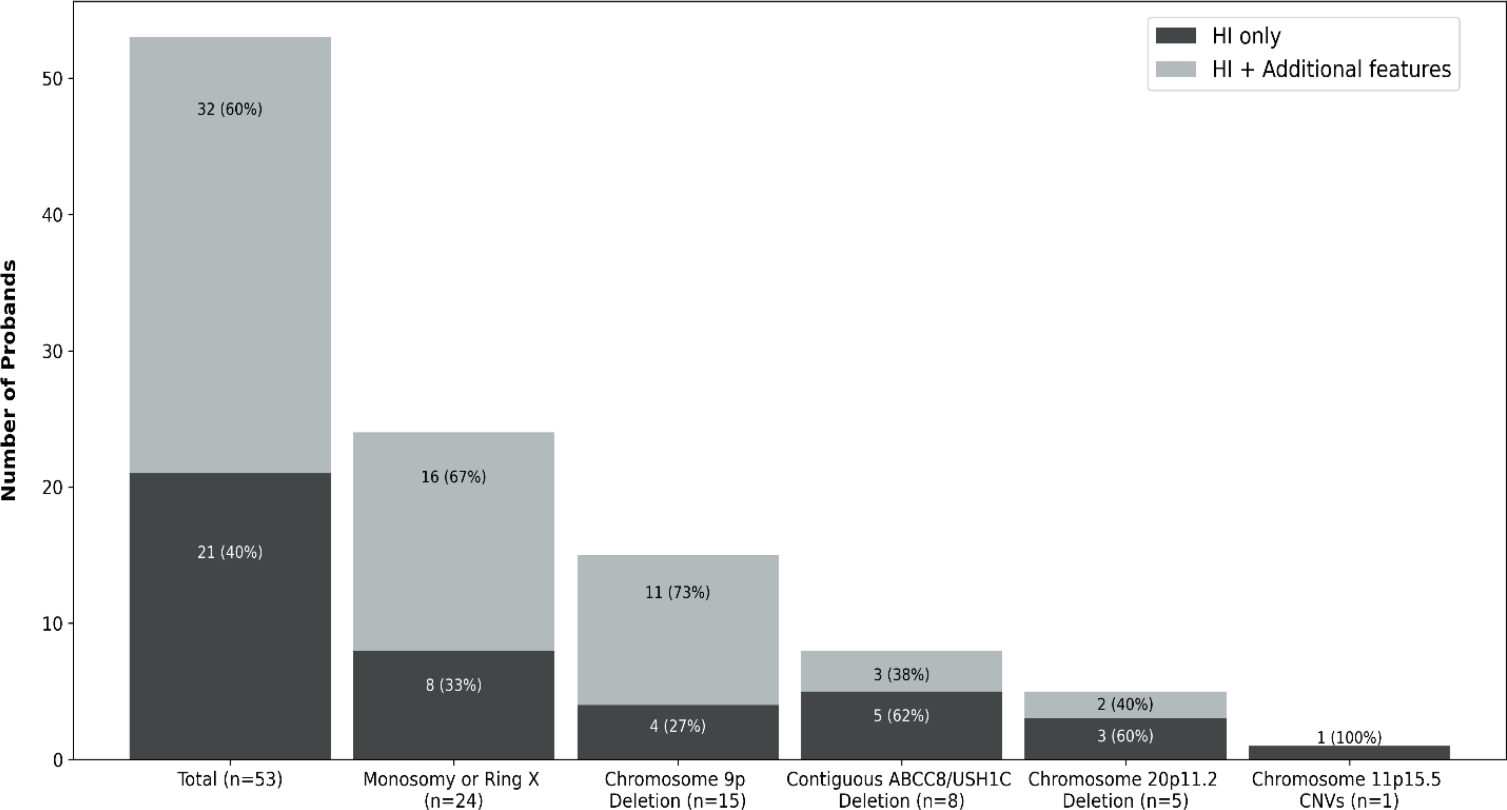
Bar chart representing the number of probands with HI resulting from contiguous gene copy number variants (CNVs) in our cohort. Each bar is separated according to reported additional clinical features at the time of referral for genetic testing for HI. Dark grey represents the number of children where HI was the only reported feature, light grey denotes the number of children where extra-pancreatic feature(s) were reported at referral.

Contiguous gene CNVs affecting 5 of the 6 genomic loci were identified in 53 probands: 28 were from the genetically solved cohort and 25 were from the genetically unsolved cohort (**Figure 1**).

We detected a contiguous gene CNV affecting the X chromosome in 24 females (24/53; 45%). Microarray analysis or karyotyping had already established a diagnosis of Turner syndrome in 14 of these children prior to referral for HI genetic testing. At least 11 individuals had a deletion that encompassed *KDM6*A (the exact coordinates of the variants were not available for 13 individuals). We identified a terminal chr9p deletion in 15 individuals which ranged in size from ~7.2Mb to 19.4Mb. Microarray analysis had detected the deletion in 6 of these individuals prior to referral for HI genetic testing. Five patients had a chr20p11.2 deletion ranging in size from ~3Mb to 8Mb. In one child, we identified a 4.8Mb duplication on the paternal allele at the chr11p.15.5 differentially methylated region, confirming a diagnosis of Beckwith-Wiedemann syndrome (15). SNP array analysis of all 8 individuals in the solved cohort with a deletion of *ABCC8* exons 1-22, demonstrated the deletion extended over exons 1-19 of the adjacent *USH1C* gene, confirming a diagnosis of Usher syndrome type 1c. We did not detect any CNVs encompassing the *NSD1* gene at chr5q35, which would have been consistent with Sotos syndrome (15). We previously reported clinical and genetic data for 9 of the 15 individuals with a chr9p deletion and the 5 individuals with a chr20p11.2 deletion (12, 14).

**Figure 2:**
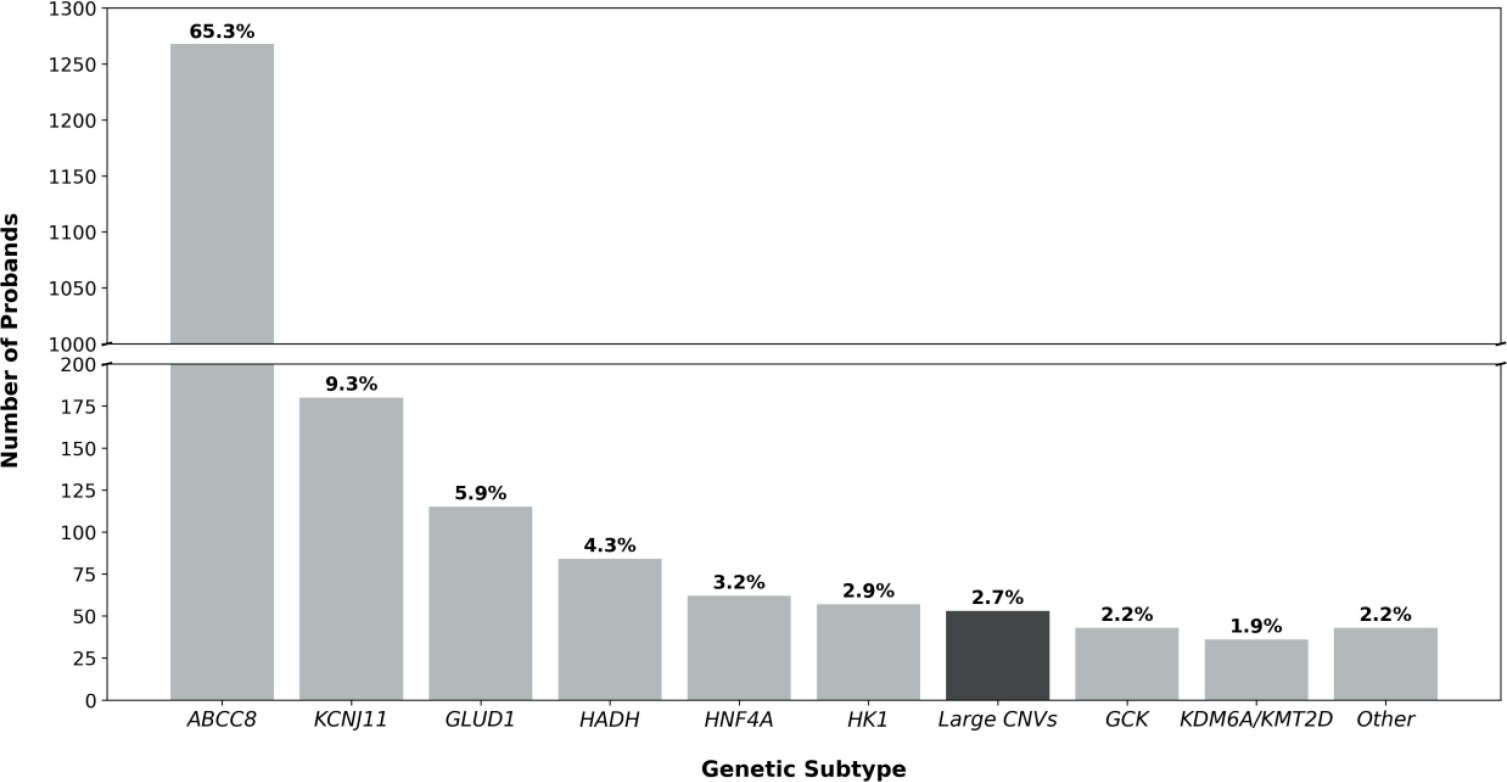
Bar chart detailing the genetic etiology of the 1941 probands in our genetically solved cohort. When the prevalence of a genetic etiology was less than 1%, genetic causes were grouped under ‘Other’ (*EP300, NSD1, FOXA2, CACNA1D, PMM2, HNF1A, INSR*). The bar highlighted in black represents the prevalence of contiguous gene copy number variants (CNVs) in the HI probands reported in this study (2.7%, 53/1941).

Identifying 25 contiguous gene CNVs in the genetically unsolved HI cohort increased the pick-up rate for disease-causing variants from 50.9% (95% CI: 49.3-52.5%, n=1,916/3,763) to 51.6% (95% CI: 50.0-53.2%, n=1,941/3,763). These variants had an overall prevalence of 2.7% (95% CI: 2.1-3.6%, n=53/1,941) in the solved cases, which is similar to the prevalence of variants in other routinely tested HI genes (**Figure 2**).

The median age at diagnosis of HI in the 53 individuals with a contiguous gene CNV was 1 day (range, 0 days – 6 years) and their median birthweight was +0.41 SDS (range, -2.3 to +3.1 SDS). We obtained treatment data for 43 individuals at referral for genetic testing. Among them, 35 children were treated with diazoxide, 3 with octreotide and 1 with a combination of diazoxide and octreotide. Four children with Turner syndrome were not receiving treatment at referral because their HI was in remission, and two children, both with Usher syndrome type 1C, had undergone near total pancreatectomy.

**Figure 3:**
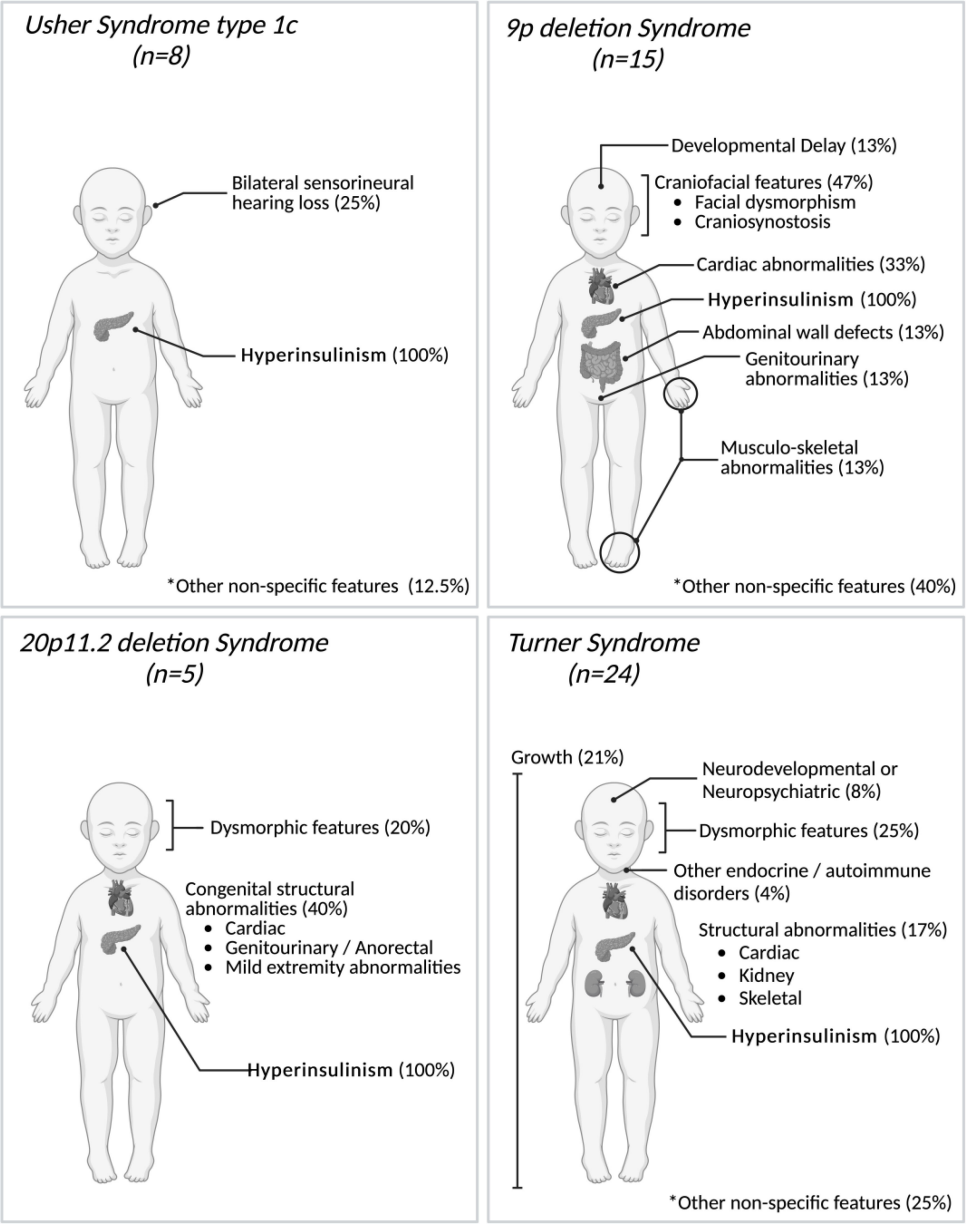
Phenotypic features reported in 52 individuals with contiguous gene copy number variants (CNVs) at the time of referral for genetic testing for HI. All reported features are summarized under generalized categories. Non-specific features refer to features that are rarely reported to be associated with these specific syndromes and/or that could be more common in the general population. Percentages are based on the number of individuals with these features with the possibility that one individual may have had several and/or non-specific features. The single case with a large duplication at the Beckwith-Wiedemann syndrome locus has not been depicted as no additional features were listed at the time of referral for genetic testing. Created in BioRender. Lazaridi, I. (2025) https://BioRender.com/e55o581.

Clinicians reported additional features in 32 of the 53 individuals (60%) at the time of referral for genetic testing (**Figure 1**). In all but two cases, at least some of the additional features reported were consistent with the genetic diagnosis, though not necessarily pathognomonic for the condition (**Figure 3**).

The median age at referral of individuals within the solved cohort (i.e. those where the contiguous gene CNV that had already been detected prior to referral for HI testing) was 409 days (range, 11–4262 days). This was higher but not significantly different to those who underwent HI genetic testing as a first-line test (median 191 days, range 12–4065 days) (p > 0.05, Mann Whitney U).

## Discussion

We comprehensively screened for contiguous gene CNVs in a large cohort of patients referred for routine HI genetic testing and identified variants in 53 individuals across five distinct genetic loci. Collectively, these variants accounted for 2.7% of genetic diagnoses, a prevalence comparable to that of variants in *HNF4A*, *HK1*, and *GCK* (3.2%, 2.9% and 2.2% respectively; **Figure 2**); genes which are routinely included on gene panels for HI.

Twenty-one probands (40%) did not have extra-pancreatic features listed on the genetic testing request form, suggesting that HI was the presenting feature of their condition (30, 31). This finding highlights the need to screen for contiguous gene CNVs in all individuals with HI, regardless of their phenotype. Detecting these variants early is important because it informs prognosis, allowing for proper monitoring and the prompt diagnosis and medical management of additional clinical features if/when they develop.

The most common contiguous gene CNVs we identified were deletions on the X chromosome. These were identified in 24 females. In at least 11 patients, the deletion extended over the HI and Kabuki syndrome gene (*KDM6A*). Haploinsufficiency of this gene has been proposed as a mechanism for the increased prevalence of HI observed in girls with Turner syndrome (20). In 14 children, clinicians noted additional features prior to referral for HI genetic testing. These had prompted karyotyping or microarray analysis as a first line genetic test which confirmed Turner syndrome. Six of the 15 probands with a chr9p deletion had also undergone microarray analysis prior to referral for HI genetic testing which detected the deletion.

There are several reasons why patients who had already received a genetic diagnosis might have been sent for additional HI genetic testing. While the first papers describing the association between HI and Turner syndrome and HI and the 9p deletion syndrome were published in 1979 and 1996 respectively (32, 33), it has only been in the last few years that the role of these contiguous gene CNVs in the etiology of HI has been firmly established (12, 20). In keeping with this, 13 of the 14 individuals with a prior diagnosis of Turner syndrome were referred for HI genetic testing before the 2018 publication by Gibson et al., which described 12 girls with HI and X chromosome abnormalities (20). Similarly, all 6 patients with previously confirmed chr9p deletions were referred prior to the 2019 publication by Banerjee et al. (12 *)*,. In addition, it was possible that some children may have had a contiguous gene CNV and co-incidental HI (digenic disease). Because of this, clinicians may have considered comprehensive HI genetic testing necessary, particularly for those children with persistent disease that responded poorly to treatment as finding a pathogenic variant in a K-ATP channel gene for example would inform medical management (34).

We identified contiguous gene CNVs in 1.4% (95% CI: 1.1%-1.8%, n=53/3,763) of our whole HI cohort. This figure likely reflects a minimum prevalence due to several referral biases. For example, our cohort has an underrepresentation of children with Beckwith-Wiedemann syndrome as this condition is often diagnosed clinically prior to confirmatory methylation testing. We also did not search for mosaic CNVs, as these would have been called with less confidence potentially resulting in false positive calls. Therefore, it is possible that we missed some clinically relevant variants especially those causing Turner syndrome, where mosaicism has been observed (20). Finally, we limited our search to include CNVs with strong evidence for their causative role in HI, other HI disease-causing CNVs will therefore not have been detected.

In this study, we screened for contiguous gene CNVs using WGS data or off-target reads generated from routine tNGS analysis. Until recently, many laboratories have employed more cost-effective targeted gene panels or exome sequencing for screening the HI genes due to the higher costs of WGS (5). While both methods can be adapted to target these contiguous gene CNVs, their large size makes them difficult to screen for using these approaches. As the cost of WGS continues to decrease, laboratories will likely adopt it as the gold standard first-line genetic test for HI, making the detection of these contiguous gene CNVs easier. Until then, we recommend considering a CNV screen when a large deletion is detected in the *ABCC8*, *KDM6A* (females only), or *FOXA2* genes, as these might indicate Usher, Turner and 20p11.2 deletion syndromes respectively.

While we have shown that our in-house software, SavvyCNV can call off-target CNVs from small tNGS panels with high precision and recall, we recognize that this methodology does not meet diagnostic standards for CNV calling (26). We used this approach in this study as it allowed for CNV analysis using existing data at no additional cost. As such, when we identified a novel contiguous gene CNV, we informed the clinicians and advised them to request orthogonal testing, which confirmed our findings in all cases.

In conclusion, we have shown that contiguous gene CNVs are an important cause of HI that should be considered by laboratories and clinicians managing children with this condition.

## Data Availability

The datasets presented in this article are not readily available to preserve patient confidentiality. Sequencing data is available through collaboration to experienced teams working on approved studies examining the mechanisms, diagnosis and treatment of diabetes and other beta cell disorders. Requests for collaboration will be considered by a steering committee following an application to the Genetic Beta Cell Research Bank (https://www.diabetesgenes.org/current-research/genetic-beta-cell-research-bank/). Contact by email should be directed to Sarah Flanagan (s.flanagan@exeter.ac.uk).
